# Effectiveness of ChatGPT and DeepSeek in Urology Medical Education: Randomized Controlled Trial

**DOI:** 10.2196/89315

**Published:** 2026-09-21

**Authors:** Wentong Yang, Ting Xu, Junjie Wei, Wentao Zheng, Wenbo Yan, Jingkai Wang, Guangdi Chu, Haitao Niu

**Affiliations:** 1 Department of Urology, The Affiliated Hospital of Qingdao University, No. 16 Jiangsu Road, Qingdao, Shandong, 266003, China, 86 186 61803117, 86 0532 82912105; 2 Department of Geratology, No. 971 Hospital of the People's Liberation Army Navy, Qingdao, Shandong, China; 3 Qingdao Medical College, Qingdao University, Qingdao, Shandong, China

**Keywords:** generative AI, ChatGPT, DeepSeek, medical education, urology, randomized controlled trial, large language models

## Abstract

**Background:**

Since its release in November 2022, generative AI (GenAI) tools, including ChatGPT, have gained widespread attention across various sectors, including medical education.

**Objective:**

This study seeks to examine the effectiveness and feasibility of GenAI tools (ChatGPT o3‑mini [OpenAI] and DeepSeek R1) in enhancing urology teaching outcomes for medical undergraduates.

**Methods:**

We assessed the accuracy of responses from ChatGPT o3-mini and DeepSeek R1 to authoritative urology multiple-choice questions. Then, a randomized controlled trial was performed to compare the learning outcomes of students using ChatGPT o3-mini and DeepSeek R1 with those using traditional learning methods. Additionally, a questionnaire was designed to survey medical undergraduates’ perspectives on the application of AI in urology education.

**Results:**

DeepSeek R1 demonstrated higher accuracy than ChatGPT o3-mini in answering urology-related multiple-choice questions. In the test following the self-study period, the DeepSeek R1 group surpassed both the control and ChatGPT o3-mini groups in total scores across various question types. Despite the superior scores in the ChatGPT o3-mini group, statistical significance was not achieved relative to the control group. Survey results revealed that most students had a positive attitude toward AI-assisted learning, believing it could effectively enhance medical education.

**Conclusions:**

DeepSeek-assisted self-study was associated with higher posttest scores than traditional internet-based learning, whereas ChatGPT showed numerically higher but nonsignificant results. These findings offer evidence-based insights into the embedding of GenAI within medical education frameworks, providing guidance for educators in developing teaching strategies and for institutions in formulating relevant policies.

## Introduction

Generative AI (GenAI), a subset of machine learning, can generate new content based on data from training sets [1]. The advent of GenAI, exemplified by ChatGPT’s (OpenAI) launch in late 2022, has sparked considerable interest in multiple fields, with medical education emerging as a key area of application [2]. Undergraduate medical education, as the cornerstone of talent development, plays a critical role in building theoretical foundations and cultivating essential clinical skills. However, it faces significant challenges. The vast and complex nature of medical knowledge requires students to master extensive information within a restricted period. Traditional teaching methods, often focused on rote memorization, result in superficial understanding, making it difficult for students to apply knowledge flexibly [3,4]. Moreover, the growing disparity in educational resources underscores the need for continuous updates to content and teaching methods to address the rapid advancements in medical technology and support students’ motivation and lifelong learning abilities [5].

As technology rapidly evolves, medical education is undergoing a profound transformation [6,7], particularly in the realm of multimedia. The development of virtual reality-assisted teaching has enriched learning experiences, enabling students to construct a deeper conceptual understanding via immersive technologies like 3D virtual anatomy software and virtual simulation laboratories [8-10]. Simultaneously, large language models (LLMs) such as ChatGPT o3-mini have been integrated into the medical education landscape as valuable aids, with potential applications in clinical decision-making [11]. GenAI models, such as ChatGPT o3-mini and DeepSeek R1, can serve as powerful supplementary tools, offering immediate answers to students’ questions and facilitating the rapid acquisition of knowledge, thus enhancing learning efficiency [12-14]. Additionally, GenAI can simulate clinical scenarios, enabling students to practice clinical decision-making and diagnostic reasoning through interactive question-and-answer sessions, effectively translating theoretical knowledge into practical clinical competence [15-17]. However, challenges persist, including concerns about the trustworthiness of AI-generated content, the potential for student overdependence on these tools, and unresolved ethical and legal issues [18,19].

Given the ongoing transformation in medical education and the advantages and challenges of GenAI in undergraduate medical education, our study aims to assess whether GenAI tools (ChatGPT o3-mini and DeepSeek R1) can enhance the teaching of urology to medical undergraduates. We quantified the performance of both models on authoritative urology multiple-choice questions (MCQs) to assess their reliability as learning aids. To evaluate the practical impact of GenAI in medical education, a randomized controlled trial was designed to compare the learning outcomes of students using ChatGPT o3-mini and DeepSeek R1 with those using traditional learning methods. Additionally, a questionnaire was designed to survey medical undergraduates’ perspectives on the application of AI in urology education, gathering student feedback to establish an evidence base for guiding the future development and application of GenAI in medical education. The findings of this study will provide an empirical foundation for the integration of GenAI into medical curricula, offering recommendations for educators in shaping teaching strategies and for institutions in formulating relevant policies.

## Methods

### Model Introduction

In this study, we selected 2 widely recognized and applicable AI models, ChatGPT o3-mini and DeepSeek R1, to evaluate their effectiveness in undergraduate urology education. ChatGPT o3-mini, an OpenAI product based on the GPT framework, derives its core competency from training on massive text datasets. This training underpins its proficiency in parsing user intent with precision and synthesizing fluent, human-like language [20,21]. The model demonstrates strong capabilities in language generation and logical reasoning, providing relevant knowledge responses and text generation services based on input queries. In the context of urology diagnosis and treatment, ChatGPT o3-mini can analyze case information, symptom descriptions, and other data to offer diagnostic suggestions, disease-related knowledge, and treatment recommendations, thereby assisting health care professionals in clinical decision-making.

DeepSeek R1, an LLM developed using deep learning techniques, was created by the Chinese company DeepSeek. Trained on vast multidomain datasets, it demonstrates excellent language comprehension and content generation capabilities. Compared to ChatGPT o3-mini, DeepSeek R1 exhibits superior accuracy and logical reasoning when addressing complex issues and specialized domain knowledge. In the context of urology diagnosis and treatment, DeepSeek R1 can analyze patient information, such as medical history, symptoms, and examination reports, to provide more precise diagnostic suggestions, differential diagnosis strategies, and personalized treatment recommendations. Its strong judgment capabilities enable it to better understand and analyze professional terminology and the complex logical relationships within medical texts.

### Accuracy Testing of Models

**Figure 1.**
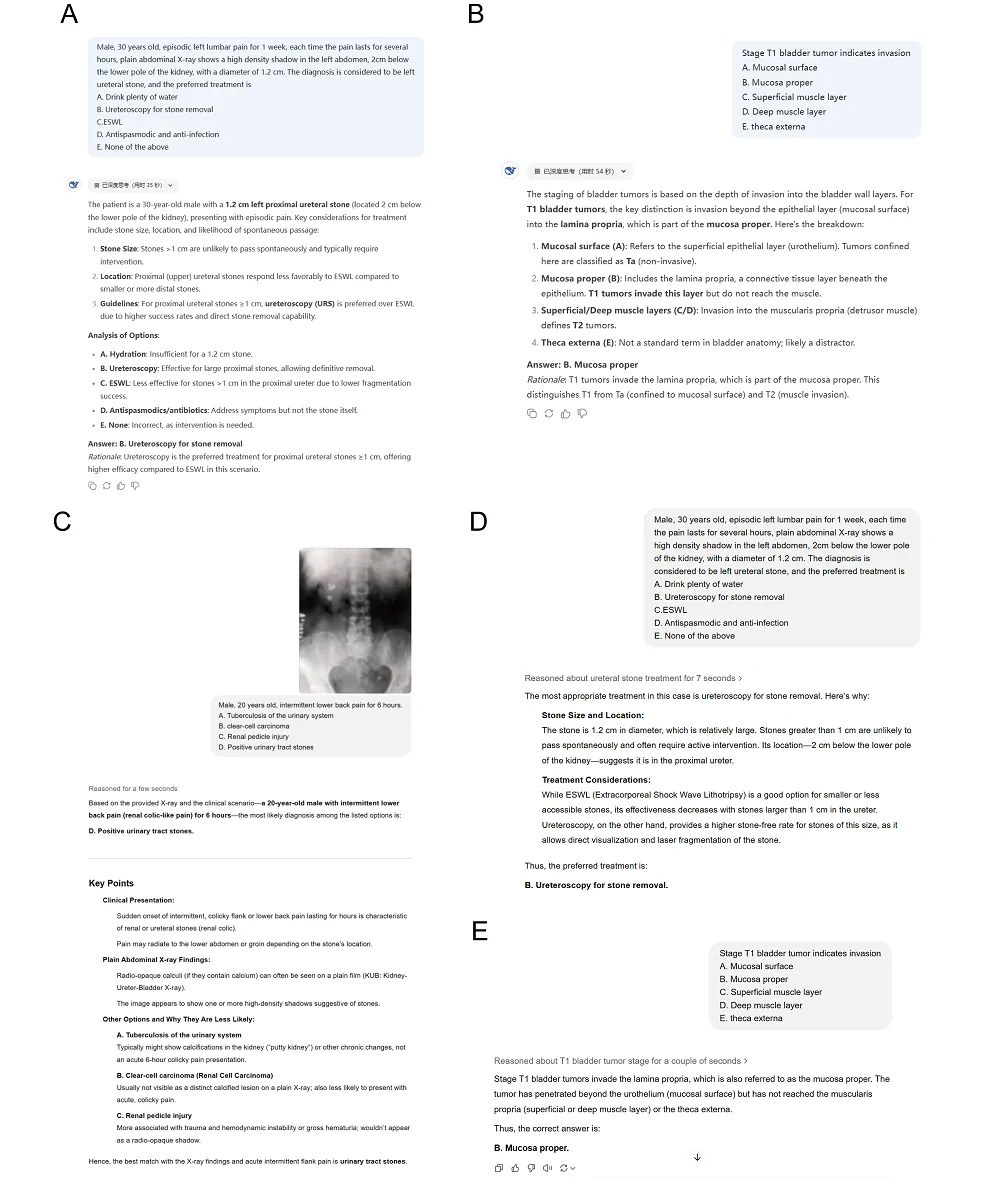
Interfaces for AI to answer questions: (A) DeepSeek R1 answering A2 type questions; (B) DeepSeek R1 answering A1 type questions; (C) ChatGPT o3-mini answering image type questions; (D) ChatGPT o3-mini answering A2 type questions; and (E) ChatGPT o3-mini answering A1 type questions.

We used 185 urology-related MCQs from the National Medical Electronic Schoolbag software (Beijing Medical Vision World Technology Co, Ltd), which were selected from the standardized residency training examination in China, to assess the accuracy of responses generated by ChatGPT o3-mini and DeepSeek R1. All model interactions occurred on March 14, 2025. Each question was presented to both ChatGPT o3-mini and DeepSeek R1 using the default chat interface. The temperature was default for both models. To ensure reproducibility of our assessment, the prompts were used (details provided in Multimedia Appendix 1) and were written in English and Simplified Chinese. Web search was available for both models. Figure 1 shows typical answer explanations generated by each model. These questions, drawn from authoritative sources within the standardized residency training examination, cover a wide range of urological knowledge, including fundamentals, diagnosis, and treatment, ensuring their representativeness and reliability. Each question was posed to the AI models 3 times, with the responses categorized into 4 levels: completely correct, more correct than incorrect, more incorrect than correct, and completely incorrect. If all 3 responses were correct, it was recorded as completely correct, earning 3 points; if 1 of the 3 responses was incorrect, it was recorded as more correct than incorrect, earning 2 points; if 2 of the 3 responses were incorrect, it was recorded as more incorrect than correct, earning 1 point; if all 3 responses were incorrect, it was recorded as completely incorrect, earning 0 points. These categories allowed for a quantitative assessment of the accuracy of the AI-generated responses [22].

In the comparative analysis of ChatGPT o3-mini and DeepSeek R1, the 3 responses generated by each model for every question were aggregated, with a score of 3 considered a correct answer to assess the stability of models. We then calculated the overall accuracy and average scores for both models across all questions under identical conditions to assess differences in performance. Additionally, accuracy rates and average scores were calculated for various question categories (such as anatomy, diagnosis, treatment, etc) to compare the models’ performance across domains, providing a comprehensive assessment of their accuracy and strengths in urology-related medical knowledge.

### Sample Size Estimation, Participant Characteristics, and Control Group Design

This randomized controlled trial was conducted in accordance with the CONSORT-EHEALTH (Consolidated Standards of Reporting Trials of Electronic and Mobile Health Applications and Online Telehealth) checklist and guideline (Checklist 1). For the sample size calculation comparing the means among 3 independent groups, we set the statistical power (1-β) at 80% (power=0.80) and the significance level (α) at 0.05. The trial was designed with 3 groups (k=3) and an allocation ratio of 1:1:1. The assumptions for means and SDs were determined with reference to the data distributions reported in 2 recent comparable studies. Wu et al [23] used a Chinese question bank for hepatobiliary surgery internship teaching, where the traditional group had an average theoretical score of 77.86 (SD 4.16) and the ChatGPT o3-mini group scored 86.44 (SD 5.59). Wang et al [24] conducted structured clinical tests in medical history collection training, with the baseline GPT and control groups scoring 57.39 (SD 11.14) and 54.68 (SD 10.33), respectively. Based on these data, we conservatively estimated the average score for the control group to be 75 (SD 10) and anticipated that the 2 AI groups (ChatGPT o3-mini and DeepSeek R1) would achieve an average score of 85 (SD 11). Based on a 20% dropout rate, the sample size for each group estimated by PASS (Power Analysis and Sample Size) 2021 (NCSS, LLC) was 76, resulting in a total of 228 participants, which meets the statistical requirements.

A total of 228 undergraduate students from the Medical College of Qingdao University were included in this study, ranging from the second to the fifth year. The inclusion criteria were that they were majoring in clinical medicine and had completed courses such as systemic anatomy, regional anatomy, histology, embryology, and biochemistry, with no failing records. The exclusion criteria included students who had failed courses, changed majors, used AI in the control group, or refused to participate or dropped out for personal reasons.

### Randomization

This study used a parallel-design, prospective randomized controlled trial. The allocation was indeed designed as 1:1:1. We used a sealed envelope randomization method: a total of 240 sealed envelopes were prepared, with 80 envelopes allocated to each of the 3 groups (ChatGPT, Control, and DeepSeek), reflecting the intended 1:1:1 allocation ratio. Among the 228 eligible students recruited for the study, 8 declined to participate, leaving 220 participants who were randomly assigned to 1 of the 3 groups by drawing 1 sealed envelope each. All participants were assigned by randomly drawing sealed, opaque, sequentially numbered envelopes prepared in advance with a 1:1:1 allocation ratio. The envelope opening and group assignment were conducted by an independent research coordinator who was not involved in participant recruitment or outcome assessment, ensuring no manual intervention in group assignment, no selective enrollment, and no randomization errors. The 2 models were applied to the ChatGPT o3-mini and DeepSeek R1 groups as supplementary learning tools, aiming to explore their effects and roles in helping medical undergraduates master urology knowledge. Additionally, through tests and questionnaires, we assessed students’ views and experiences regarding the application of these models in urology education.

### Learning Materials

In this study, the learning materials for participants were drawn from the National Comprehensive Cancer Network (NCCN) guidelines, the European Association of Urology (EAU) guidelines, and Chinese specialized textbooks used in higher medical education. Specifically, the study materials covered a range of urological disease types, including urinary stone disease and tumors, thereby comprehensively covering core knowledge domains in urology (Table 1). These resources, chosen for their authority and scientific rigor, underwent a rigorous selection and integration process to ensure both accuracy and current relevance. The NCCN and EAU guidelines serve as prominent international references in the field of urology and provide up-to-date diagnostic and therapeutic paradigms, whereas the Chinese medical education textbooks are more closely aligned with the participants’ domestic educational background and local clinical context. The combined use of these resources was designed to present participants with a systematic, comprehensive, and pragmatic body of knowledge, enabling a deeper understanding and mastery of key urology concepts and thereby laying a solid theoretical foundation for their future clinical practice.

**Table 1.** Urology learning content selected for the randomized controlled trial.

Chapter and section	Item
Main symptoms of urinary system	
Symptoms related to urination	Classification of urinary incontinence Localization analysis of hematuria
Injury of urinary system	
Closed renal injury	Classification Treatment
Urethral injury	Classification and causes of injury Diagnosis Treatment
Bladder injury	Classification and causes of injury Injury types Diagnosis
Obstruction of urinary system and urolithiasis	
Prostate hyperplasia	Clinical manifestation Diagnosis Treatment
Bladder stones	Clinical manifestation Diagnosis Treatment
Renal and ureteral stone	Clinical manifestation Diagnosis Treatment
Tuberculosis of urinary system	
Renal tuberculosis	Etiology and pathology Clinical manifestation Diagnosis Treatment
Tumors of urinary system	
Renal tumor	Renal carcinoma Nephroblastoma Renal pelvic carcinoma
Tumors of urinary system	
Bladder cancer	Etiology and pathology TNM classification Clinical manifestation Diagnosis Treatment

### Group Design

This study used a parallel-design randomized controlled trial to assess the impact of ChatGPT o3-mini and DeepSeek R1 on learning outcomes in urology undergraduate education. Participants were stratified and randomized by grade level into 3 groups: the ChatGPT o3-mini group, the DeepSeek R1 group, and the control group. Participant allocation was performed using a grade-level stratified randomization approach combined with grouping based on prior academic performance, ensuring an equal grade distribution across the groups. Strictly unified inclusion and exclusion criteria were applied; all enrolled students had completed the core foundational medical courses with no course failures and demonstrated comparable overall academic proficiency levels. All learning materials for the participants were sourced from the materials mentioned earlier. In the ChatGPT o3-mini and DeepSeek R1 groups, students were required to use the specified AI tools (ChatGPT o3-mini or DeepSeek R1) to search for knowledge to support their learning and were not allowed to use other internet search engines. They could ask the AI questions based on the daily review content to obtain explanations of urology-related knowledge and learning suggestions in order to help deepen their understanding and memory of the review materials. To ensure optimal learning effects for participants in the experimental groups using AI tools, group coordinators collected daily usage durations through interactive conversations, guaranteeing that each participant spent at least 30 minutes per day engaging with the AI system. In this study, if students had questions or difficulty understanding AI-generated content, they could consult urologists who were invited by the researchers for clarification. Additionally, to avoid bias arising from differences in individual learning habits that may affect outcomes, we explicitly required participants in the AI groups to use identical prompt phrases. In contrast, the control group was limited to rely exclusively on traditional internet search methods, such as forums and search engines, to find relevant information for their review and was prohibited from using any AI-related software. To explain how the varying dropout rates affect the results and validity, we performed an intention-to-treat (ITT) analysis using 2 methods: worst-case imputation and multiple imputation.

### Follow-Up Strategy

The test content was derived from previous questions of both the Chinese National Medical Practitioner Examination and the United States Medical Licensing Examination and was designed to be completed within 2 hours, with a maximum score of 100 points. After the test, 2 researchers independently reviewed and scored the responses within 20 days to maintain consistency and impartiality in scoring. The primary end point of this study is total test scores of the 3 groups. The remaining measures (the scores of the 3 groups on type A1 and type A2 questions, image type questions, and text type questions) are secondary or exploratory end points. The 2 researchers who independently scored the multiple-choice tests were blinded to participants’ group allocation. Specifically, all participant identities were anonymized and recorded using numeric participant IDs only, with no information about whether the participant belonged to the DeepSeek R1, ChatGPT o3-mini, or control group. The group allocation key was kept separately by a third researcher who was not involved in scoring. Therefore, the outcome assessors were effectively blinded. Additionally, all participants in the 2 experimental groups completed a follow-up questionnaire. The design of the questionnaire was informed by relevant research literature [25,26] and used a Likert scale to assess participants’ perspectives and experiences with AI-assisted learning. The questionnaires were all voluntary to fill out, and those who left any information blank were required to fill it in again to ensure an effective completion rate. The questionnaire consisted of 6 key sections. The first section gathered baseline characteristics, including participants’ gender and grade. The second section explored AI usage, focusing on its purpose, frequency, and any challenges encountered during use. The third section assessed participants’ knowledge of AI, specifically regarding its potential to alleviate the burden on both teachers and students, its ease of use, and its ability to enhance understanding and stimulate interest in learning. The fourth section addressed concerns about AI usage, examining participants’ trust in AI, their concerns, and their views on its standardized application. In the fifth section, the survey investigated participants’ opinions on the future integration of AI in undergraduate medical education, including its potential to transform teaching methods, replace educators, address resource imbalances, and integrate with traditional education models. Finally, the sixth section solicited suggestions from participants on how AI could be more effectively integrated into undergraduate medical education.

### Statistical Analysis

The age and gender of respondents and nonrespondents were compared using the 2-tailed independent samples *t* test and chi-square test, respectively. The survey questionnaire used frequency and percentage distributions to describe the experimental groups’ views and experiences regarding AI-assisted learning. The results of the multiple-choice test were expressed as mean (SD) to represent each group’s performance across various question types and learning outcomes, including total scores, A1 category question scores, A2 category question scores, imaging-based question scores, and text-based question scores. Data analysis was performed using GraphPad Prism (version 9.1.0; GraphPad Software) and R (version 4.4.1; R Core Team). As the data violated the assumption of normality, comparisons among the 3 groups (Control, ChatGPT, and DeepSeek) were performed using the Kruskal-Wallis test. When the overall test was significant (*P*<.05), pairwise comparisons were conducted by Dunn post hoc test with a Bonferroni correction for multiple comparisons. Effect size was quantified using ε², with 95% CIs calculated via bias-corrected and accelerated bootstrap with 1000 resamples. A *P* value of less than .05 was considered statistically significant.

### Primary and Sensitivity Analyses

The primary analysis was performed using ITT principles, encompassing all randomized participants (N=228). Missing test scores for the 8 participants who did not attend the examination were imputed using multiple imputation by chained equations with predictive mean matching, generating 20 imputed datasets under the missing-at-random (MAR) assumption (using the mice package in R, version 4.4.1; seed=123). To evaluate the robustness of findings, 3 sensitivity analyses were conducted: (1) a complete-case analysis, including only participants with complete outcome data (n=220); (2) a worst-case analysis, where missing values were replaced with the minimum observed score within each respective group; and (3) a best-case analysis, where missing values were replaced with the maximum observed score within each group. All analyses used linear regression with group assignment as the independent variable.

### Ethical Considerations

This study was approved by the Ethics Committee of Affiliated Hospital of Qingdao University (QYFYWZLL30898), and it was also registered with the Chinese Clinical Trials Registry (ChiCTR2600118749). The Ethics Committee of the Affiliated Hospital of Qingdao University reviewed the study protocol to ensure that participants’ personal information would not be disclosed. In accordance with the Declaration of Helsinki [27], written informed consent was obtained from all participants prior to the collection of any information pertaining to them.

## Results

### Participants

**Figure 2.**
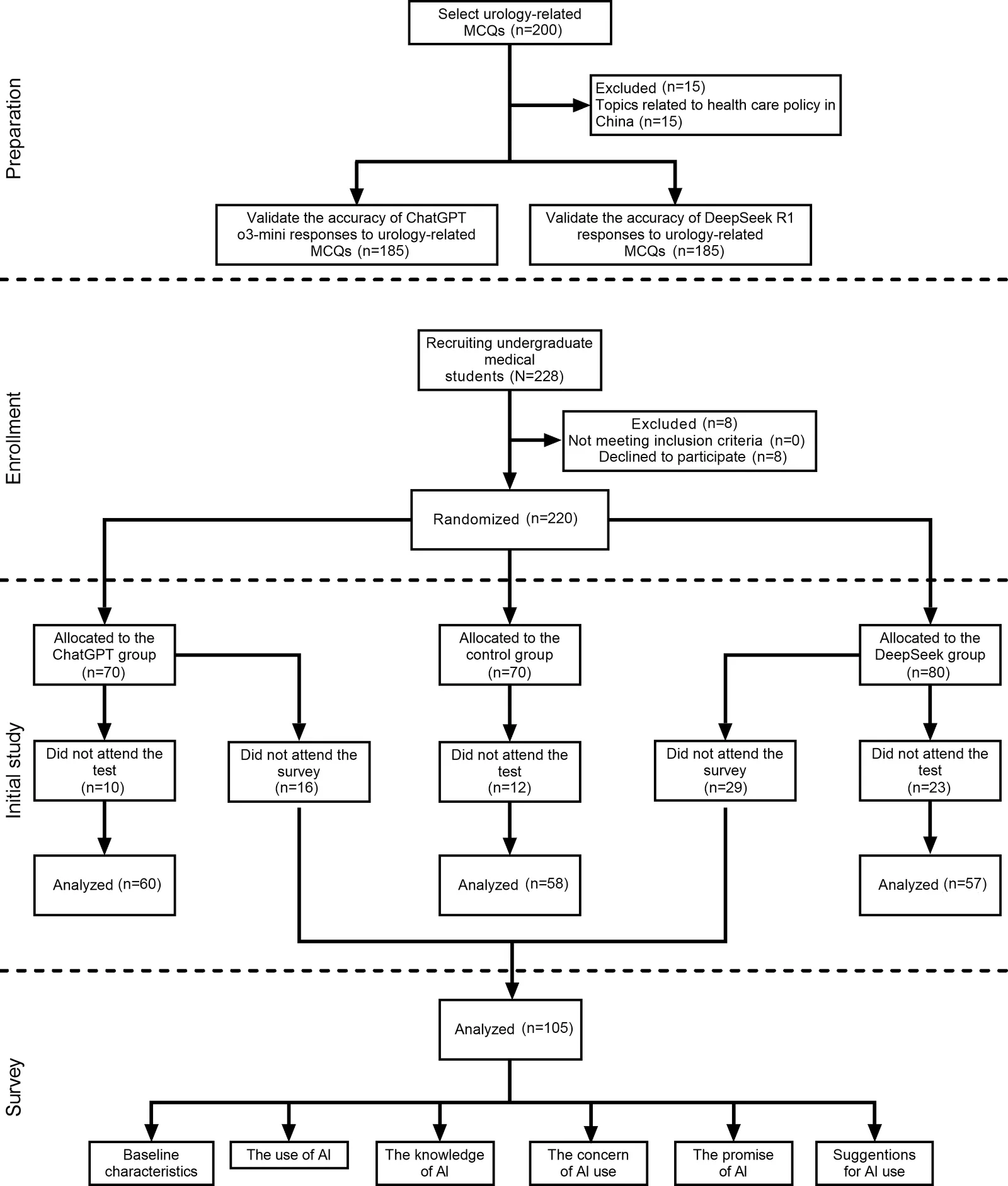
Study design and flow chart. MCQs: multiple-choice questions.

As of March 9, 2025, a total of 228 undergraduate students from Qingdao University Medical College were recruited to participate in the experiment. However, 8 participants withdrew before the randomization process. Additionally, 45 students from the 3 groups did not complete the multiple-choice test. Therefore, by April 5, 2025, a total of 175 students had completed the multiple-choice test (Table 2). Furthermore, 45 students from the 2 AI groups did not participate in the questionnaire survey. As a result, by April 5, 2025, a total of 105 students had completed the survey. The flowchart depicting the experimental procedure is presented in Figure 2.

**Table 2.** Baseline characteristics and test scores of medical undergraduates participating in the test.

Characteristic	ChatGPT o3-mini group	DeepSeek R1 group	Control group
Age (y), mean (SD; range)	20.68 (1.32; 19‐22)	20.70 (1.27; 19‐22)	20.64 (1.36; 19‐22)
Sex (male), n/N (%)	26/60 (43.33)	29/57 (50.88)	30/58 (51.72)
Average score, mean (SD; range)	58.67 (27.62; 12‐98)	66.14 (23.25; 10‐98)	51.83 (28.02; 14‐98)

### Model Characteristics and Test Questions

Table 3 summarizes the characteristics of DeepSeek R1 and ChatGPT o3-mini. Both models are based on the Transformer architecture [28,29], but they exhibit distinct strengths shaped by their architectural designs and application foci. DeepSeek R1 focuses on cost-effectiveness, transparency, and specialized reasoning, while ChatGPT o3-mini prioritizes wide coverage, polished natural language output, and user-friendly conversations [30].

**Table 3.** Baseline characteristics and test scores of respondents and nonrespondents on the test.

Characteristic	Respondents	Nonrespondents	*P* value
Age (y), mean (SD; range)	20.67 (1.31; 19‐22)	20.53 (1.14; 19‐22)	.46
Sex (male), n/N (%)	85/175 (48.57)	31/53 (58.49)	.21

We selected 185 urology-related MCQs to assess the accuracy of responses from both the ChatGPT o3-mini model and the DeepSeek R1 model. These questions were categorized into various fields: anatomy (9 questions), diagnosis (84 questions), treatment (51 questions), etiology (5 questions), clinical manifestations (9 questions), complications (5 questions), and concepts (22 questions, which did not fit into the other categories). The accuracy rates for each category, as well as the overall accuracy rate, were calculated and analyzed.

### Model Response Accuracy

In the test consisting of 185 urology-related MCQs, ChatGPT o3-mini and DeepSeek R1 exhibited different accuracy performances (Table 4).

**Table 4.** Comparison of technical characteristics between DeepSeek R1 and ChatGPT o3-mini.

Comparison feature	DeepSeek R1	ChatGPT o3-mini
Core architecture	Open-source; rule-based reinforcement learning without pre-SFT^a^ [30].	Proprietary; closed-source architecture [31].
Content quality	Concise, structured; stronger completeness and currency in disease education [32].	Detailed, expressive; higher clarity and accessibility for lay audiences [33].
Privacy and deployment	Supports offline deployment [34]; customizable via open-source datasets [35].	Restricted by closed-source limits; challenges in health care privacy compliance [36].
Key application strengths	Clinical decision support, specialized medical education [32,37].	General patient communication, broad medical education, cross-domain usability [38].

a SFT: supervised fine‑tuning.

**Figure 3.**
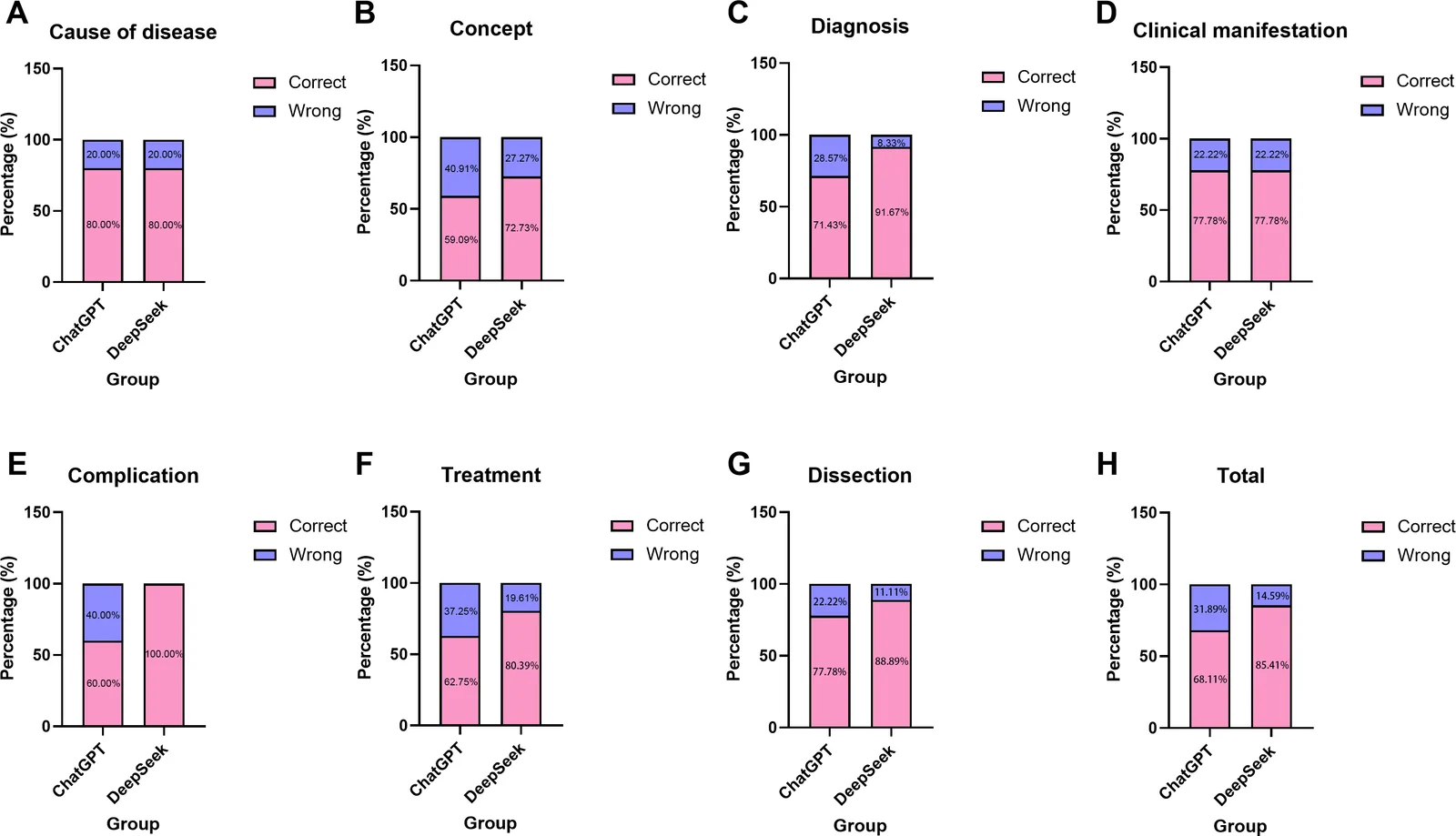
The accuracy rates of AI responses to urology multiple-choice questions and the proportion of each type of questions. Comparison between the accuracy rate of ChatGPT o3-mini and DeepSeek across (A) “cause of disease”, (B) “concept”, (C) “diagnosis”, (D) “clinical manifestation”, (E) “complication”, (F) “treatment”, (G) “dissection”, and (H) ”total” question categories.

Figure 3 presents the accuracy rates of the 2 models for each question type and overall, as well as the proportion of each question category (Table 5). ChatGPT o3-mini attained an overall accuracy rate of 68.11% (126/185). The accuracy rates for specific question types were as follows: etiology (4/5, 80%), concepts (13/22, 59.09%), diagnosis (60/84, 71.43%), clinical manifestations (7/9, 77.78%), complications (3/51, 60%), treatment (32/51, 62.75%), and anatomy (7/9, 77.78%). In contrast, DeepSeek R1 achieved an overall accuracy rate of 84.32% (156/185). The accuracy rates for specific question types were as follows: etiology (4/5, 80%), concepts (15/22, 68.18%), diagnosis (76/84, 90.48%), clinical manifestations (7/9, 77.78%), complications (5/5, 100%), treatment (41/51, 80.39%), and anatomy (8/9, 88.89%). DeepSeek R1 demonstrated higher accuracy across all domains compared to ChatGPT o3-mini, with particularly significant advantages in the areas of diagnosis, complications, and treatment.

**Table 5.** Accuracy of DeepSeek R1 and ChatGPT o3-mini on 185 urology multiple-choice questions by different types.

Response category	Cause of disease	Concept	Diagnosis	Clinical manifestation	Complication	Treatment	Anatomy	Total
Number of questions, N	5	22	84	9	5	51	9	185
ChatGPT o3-mini, n (%)								
Completely correct (3 points)	4 (80)	13 (59.09)	60 (71.43)	7 (77.78)	3 (60)	32 (62.75)	7 (77.78)	126 (68.11)
More correct than incorrect (2 points)	0 (0)	5 (22.73)	5 (5.95)	0 (0)	0 (0)	4 (7.84)	2 (22.22)	16 (8.65)
More incorrect than correct (1 points)	0 (0)	1 (4.55)	6 (7.14)	1 (11.11)	0 (0)	5 (9.80)	0 (0)	13 (7.03)
Completely incorrect (0 points)	1 (20)	3 (13.64)	13 (15.48)	1 (11.11)	2 (40)	10 (19.61)	0 (0)	30 (16.22)
Score, mean (SD)	2.40 (1.34)	2.27 (1.08)	2.33 (1.14)	2.44 (1.13)	1.80 (1.64)	2.14 (1.23)	2.78 (0.44)	2.29 (1.15)
DeepSeek R1, n (%)								
Completely correct (3 points)	4 (80)	15 (68.18)	76 (90.48)	7 (77.78)	5 (100)	41 (80.39)	8 (88.89)	156 (84.32)
More correct than incorrect (2 points)	0 (0)	1 (4.55)	0 (0)	0 (0)	0 (0)	0 (0)	0 (0)	1 (0.54)
More incorrect than correct (1 points)	0 (0)	0 (0)	0 (0)	0 (0)	0 (0)	0 (0)	0 (0)	0 (0)
Completely incorrect (0 points)	1 (20)	6 (27.27)	8 (9.52)	2 (22.22)	0 (0)	10 (19.61)	1 (11.11)	28 (15.14)
Score, mean (SD)	2.40 (1.34)	2.14 (1.36)	2.71 (0.89)	2.33 (1.32)	3.00 (0)	2.41 (1.20)	2.67 (1.00)	2.54 (1.08)

### Test After Self-Study

In this study, the test after self-study comprised 50 MCQs, which included 5 (10%) imaging-based questions, 26 (52%) A1-type questions, and 19 (38%) A2-type questions. A1-type questions, which were the most numerous, focused on fundamental urological knowledge to ensure students had a solid grasp of core concepts. A2-type questions assessed students’ ability to analyze clinical scenarios and solve problems. Although imaging-based questions accounted for a smaller proportion, they focused on evaluating students’ skills in interpreting imaging data, a crucial aspect of diagnosing and treating urological diseases. Overall, the test was designed to assess both theoretical knowledge and clinical practice skills, with a progressive structure that moved from basic principles to clinical application. This approach effectively aligned with the educational goal of integrating theory with practice in medical education.

**Figure 4.**
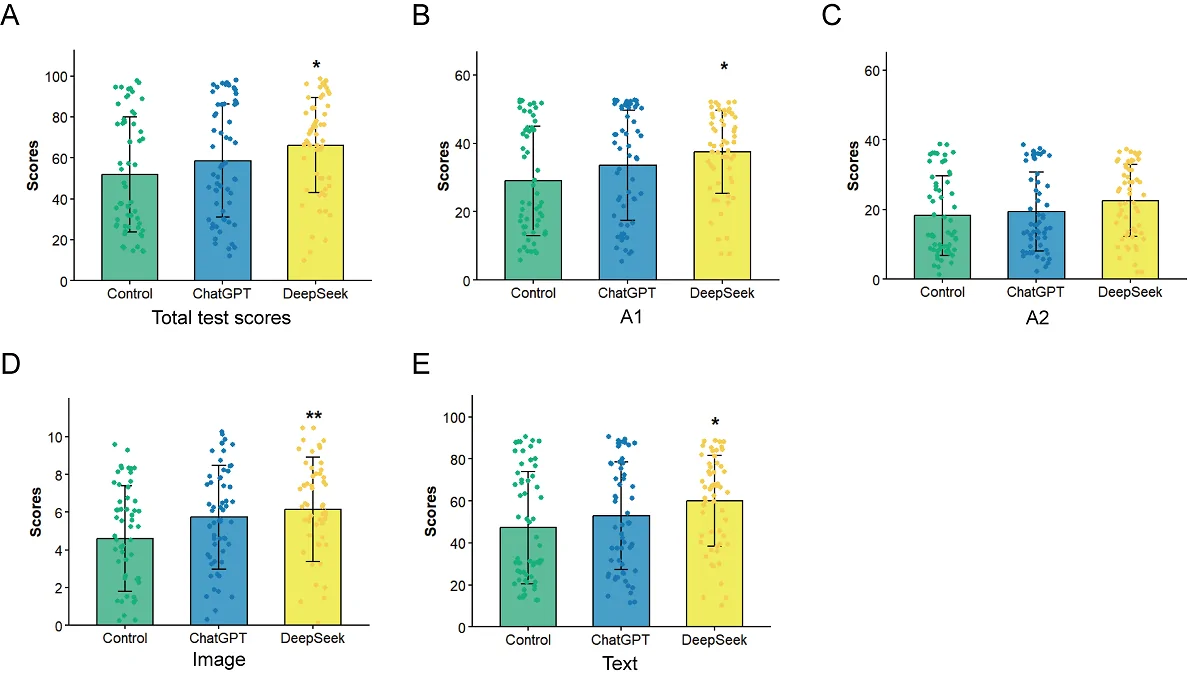
Test results across the 3 groups: (A) the total test scores of the 3 groups; (B) the scores of the 3 groups on type A1; (C) the scores of the 3 groups on type A2; (D) the scores of the 3 groups on image type questions; (E) the scores of the 3 groups on text type questions. *P<.05; **P<.01.

The test scores of each participant across the 3 groups were recorded, and the aggregate results are shown in Figure 4. Kruskal-Wallis analysis demonstrated significant differences in total test scores among the 3 models (H=7.38; *P*=.03; ε²=0.03; 95% CI 0.00-0.10; small effect). Post hoc pairwise comparisons by the Dunn test with Bonferroni correction revealed that the DeepSeek R1 group scored significantly higher than the control group (*P*=.02; Table 6). However, neither the comparison between ChatGPT and DeepSeek R1 nor that between ChatGPT and control reached statistical significance. For A1-type questions, a significant difference was likewise detected among the 3 groups (H=6.45; *P*=.04; ε²=0.03; 95% CI 0.00-0.09; small effect), with DeepSeek R1 outperforming the control group (*P*=.04) in post hoc analysis. No other pairwise comparisons were significant. Regarding A2-type questions, the Kruskal-Wallis test yielded no significant difference across the 3 models (H=4.85; *P*=.09; ε²=0.02; 95% CI 0.00-0.07; small effect). For imaging-based questions, significant intergroup differences emerged (H=9.19; *P*=.01; ε²=0.04; 95% CI 0.00-0.11; small effect). Subsequent Dunn-Bonferroni testing indicated that DeepSeek R1 achieved higher scores than the control group (*P*=.009), whereas neither the ChatGPT vs DeepSeek R1 nor the ChatGPT vs control comparison was statistically significant. Similarly, for text-based questions, the 3 models differed significantly (H=6.49; *P*=.04; ε²=0.03; 95% CI 0.00-0.09; small effect), with DeepSeek R1 surpassing the control group (*P*=.03) in pairwise comparisons; again, no significant differences were found between ChatGPT and the other 2 groups.

**Table 6.** Results of primary and sensitivity analyses.

Analysis and comparison	Estimate (95% CI)	*P* value
Intention-to-treat		
ChatGPT vs Control	6.45 (–3.74 to 16.65)	.21
DeepSeek vs Control	12.65 (0.28 to 25.01)	.045
DeepSeek vs ChatGPT	5.94 (–6.82 to 18.70)	.35
Complete-case analysis		
ChatGPT vs Control	6.84 (–2.76 to 16.44)	.16
DeepSeek vs Control	14.31 (4.59 to 24.04)	.004
DeepSeek vs ChatGPT	7.47 (–2.17 to 17.12）	.13
Worst-case scenario imputation		
ChatGPT vs Control	6.20 (–4.06 to 16.47)	.24
DeepSeek vs Control	4.20 (–5.74 to 14.14)	.41
DeepSeek vs ChatGPT	–2.00 (–11.90 to 7.90)	.69
Best-case scenario imputation		
ChatGPT vs Control	5.10 (–4.27 to 14.47)	.29
DeepSeek vs Control	16.11 (7.04 to 25.19)	.001
DeepSeek vs ChatGPT	11.01 (1.97 to 20.05)	.02

### Results of Primary and Sensitivity Analyses

DeepSeek R1 demonstrated significantly higher total scores than the control group (β=12.65, 95% CI 0.28-25.01, *P*=.045), whereas the difference between ChatGPT and control did not reach significance (β=6.45, 95% CI –3.74 to 16.65, *P*=.21). The direct comparison between DeepSeek R1 and ChatGPT also showed no significant difference in the ITT analysis (β=5.94, 95% CI –6.82 to 18.70, *P*=.35), suggesting that the observed advantage of DeepSeek R1 was primarily driven by its superiority over the control group rather than by a measurable incremental benefit over ChatGPT. Under the worst-case scenario imputation, the DeepSeek R1 advantage over control was attenuated and became nonsignificant (β=4.20, 95% CI –5.74 to 14.14, *P*=.41), whereas under the best-case scenario, the difference was strengthened (β=16.11, 95% CI 7.04-25.19, *P*<.001). For the DeepSeek R1 vs ChatGPT comparison, the worst-case scenario showed no significant difference (β=–2.00, 95% CI –11.90 to 7.90, *P*=.69), whereas the best-case scenario yielded a significant difference favoring DeepSeek R1 (β=11.01, 95% CI 1.97-20.05, *P*=.02). These findings suggest that the observed DeepSeek R1 advantage over control is maintained under standard MAR assumptions but may be partially sensitive to extreme nonrandom missingness patterns, whereas the comparison between the 2 AI models remains inconclusive regardless of missing data assumptions.

### Questionnaire Results

**Figure 5.**
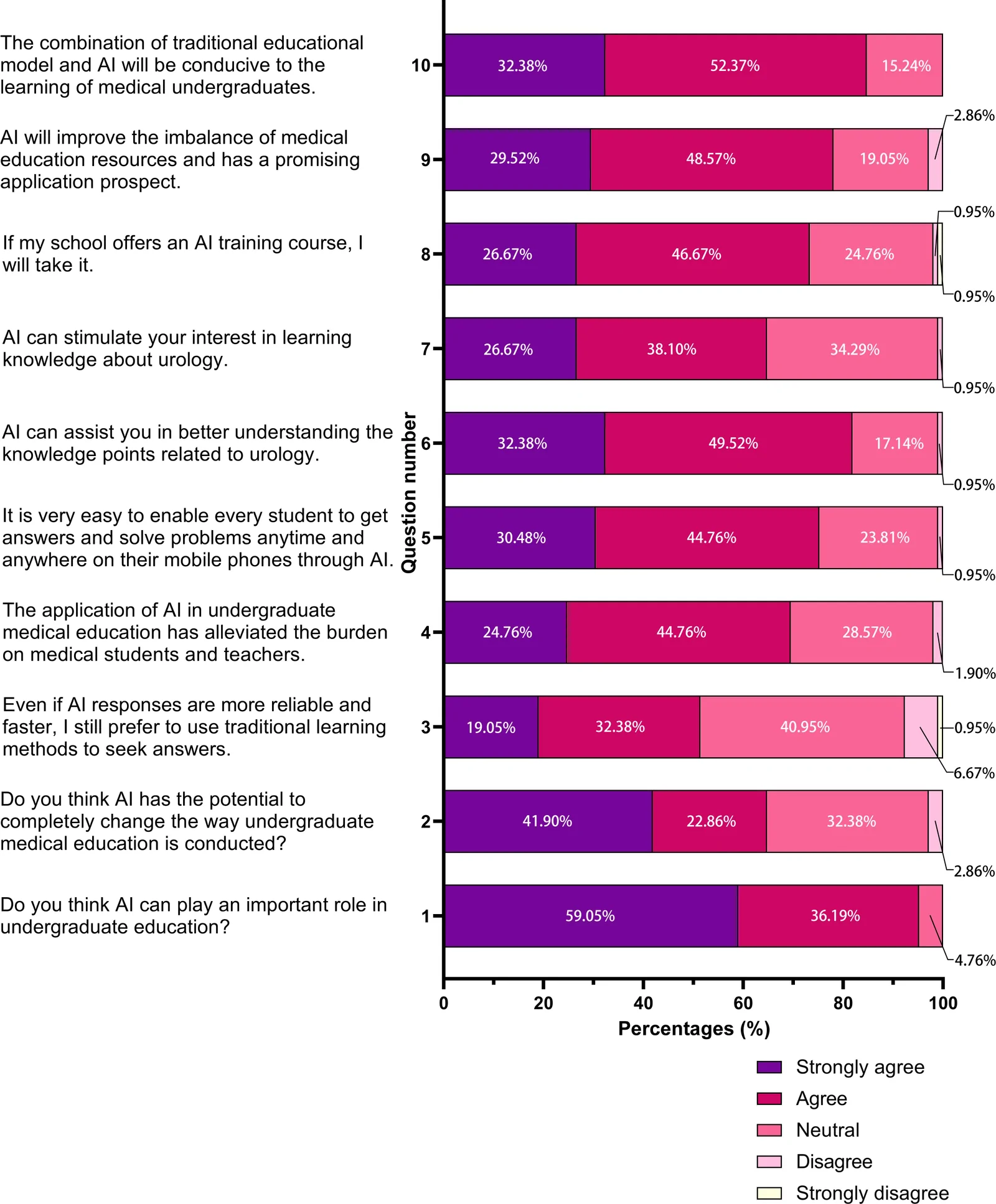
Undergraduate perceptions of the role of generative AI in medical education (n=105).

The questionnaire collected responses from 105 students about their perceptions of AI-assisted learning, and the main findings are presented in Figure 5. Regarding AI usage, most participants (n=101, 96.19%) reported using AI during their undergraduate studies. The frequency of use of GenAI by participants is shown in Multimedia Appendix 2. On the topic of AI’s role in reducing the burden on both teachers and students, 44.76% (n=47) agreed and 24.76% (n=26) strongly agreed. As for AI’s convenience, 44.76% (n=47) agreed that it allows them to seek answers anytime and anywhere, while 30.48% (n=32) strongly agreed. Furthermore, 49.52% (n=52) felt that AI helped them better understand urology-related knowledge, and 32.38% (n=34) strongly agreed. Additionally, 38.10% (n=40) agreed that AI stimulated their interest in learning urology, with 26.67% (n=28) strongly agreeing. When exploring concerns about AI use, 51.43% (n=54) of participants reported that, even if AI responses were more reliable and faster, they still preferred traditional methods for seeking answers, while 7.62% (n=8) disagreed. Regarding AI’s prospects, all participants, except for 5 neutral responses, agreed to varying degrees that AI holds transformative potential for undergraduate medical education. Furthermore, 22.86% (n=24) agreed and 41.90% (n=44) strongly agreed that AI is capable of reshaping the prevailing model of undergraduate medical education. However, only 3.81% (n=4) believed that AI would completely supersede medical educators, while 10.48% (n=11) felt that medical educators would never be replaced by AI. When asked about participating in AI training courses offered by their institution, 73.33% (n=77) of participants expressed a willingness to attend. Additionally, 48.57% (n=51) agreed and 29.52% (n=31) strongly agreed that AI could help alleviate the imbalance in medical education resources. Finally, except for 16 neutral responses, all other participants supported, to varying degrees, the future integration of AI with traditional education models.

## Discussion

### Principal Findings

Undergraduate medical education currently faces challenges such as an overwhelming curriculum, tight schedules, and students’ high cognitive load, coupled with low motivation to study [39]. In this context, the introduction of GenAI has become essential. To date, several studies have explored the integration of AI into medical education. Singla et al [40] used the Delphi method to develop an expert consensus-based AI curriculum framework for Canadian undergraduate medical students. Meanwhile, Wang et al [24] explored the use of ChatGPT in training students’ history-taking skills. The presence of GenAI in medical education prompts a reimagining and reinterpretation of traditional roles within established pedagogy [41]. On the one hand, AI can provide personalized cases instantly, based on students’ needs [42], significantly boosting curiosity and engagement. It can also generate tailored study materials based on students’ learning progress and weaknesses, such as practice questions and notes, further enhancing outcomes [43]. On the other hand, the instant feedback feature of AI can lead to overreliance, inhibiting critical thinking and independent analysis, and may raise concerns about academic integrity [44]. Thus, AI in medical education presents a dual character [45-48], with its core value lying in assisting, rather than replacing, human educators. It requires the support of teacher guidance and ethical norms to truly empower the future training of medical talents [49,50].

The primary objective of this study was to evaluate the effectiveness and feasibility of GenAI (ChatGPT o3-mini and DeepSeek R1) in enhancing medical undergraduates’ learning outcomes in urology. The results indicated that DeepSeek R1 had a higher accuracy rate than ChatGPT o3-mini in answering urology-related MCQs. In the test following the self-study period, the DeepSeek R1 group outperformed the control group and the ChatGPT o3-mini group, achieving higher total scores across various question types. Despite the elevated scores in the ChatGPT o3-mini cohort, statistical significance was not achieved relative to the control. Survey results showed that most students held a positive attitude toward AI-assisted learning, believing it effectively supports medical education. Currently, the urological community perceives ChatGPT and LLMs as promising tools for research [25], particularly given ChatGPT’s more empathetic responses [16], but remains circumspect about associated ethical challenges and the degree of patient acceptance [25].

During the study, 45 participants across all 2 groups did not take the multiple-choice test, with the majority (23 individuals) from the DeepSeek R1 group. As the control group was restricted to traditional internet search methods, which offered less novelty, participation willingness was further reduced. The students who missed the test may have had weaker academic foundations or lower motivation, potentially leading to an overestimation of the AI groups’ effectiveness. Additionally, 45 participants from the 2 AI groups did not complete the questionnaire. The high dropout rate in the questionnaire process was related to the lengthy time required for the Likert scale items and concerns about privacy in AI use. Those who did not complete the survey were more likely to hold neutral or negative views, introducing a positive bias in AI satisfaction estimates.

In terms of model accuracy, ChatGPT o3-mini showed lower accuracy on treatment and diagnosis-type questions, with rates of 62.75% (32/51) and 71.43% (60/84), respectively—significantly lower than DeepSeek R1’s performance. This suggests that ChatGPT o3-mini is not yet suitable as an auxiliary learning tool for treatment knowledge, but it is more appropriate for teaching fundamental medical concepts [51]. While AI’s diagnostic performance has not yet reached expert-level accuracy, recent research indicates that, if its limitations are well understood, AI may bring positive changes to medical education [52]. ChatGPT o3-mini’s overall accuracy rate of 68.11% (126/185) is comparable to the 70.60% MCQ accuracy reported for ChatGPT 4.0 in an English-language orthopedic question bank [51] but still lower than DeepSeek R1’s 84.32% (156/185). One possible explanation for this discrepancy is the language setting of this study, in which all questions were presented in Chinese. Since ChatGPT’s training corpus predominantly consists of English content, its ability to accurately capture the logic and semantics of Chinese medical texts may be limited [53,54]. In contrast, the DeepSeek R1 model, developed by the Chinese company DeepSeek, has been specifically optimized for Chinese language processing and demonstrates stronger performance in handling medical terminology and complex reasoning in Chinese. This language difference may help explain the performance gap observed between ChatGPT o3-mini and DeepSeek R1 in this study, particularly in Chinese-language medical education settings. Future studies should systematically compare direct Chinese queries with English queries to better understand the effect of language on model performance. Such findings would help educators decide which prompt language to recommend to students when they obtain medical information from AI.

AI-assisted learning has a significant positive impact on student performance. The DeepSeek R1 group performed exceptionally well in the test following the self-study period, indicating effective support for their learning. In A1-type questions, the DeepSeek R1 group scored 37.44 points, significantly higher than the control group’s 29 points (*P*=.01). Although students using AI tools demonstrated superior performance on A2-type questions, this advantage did not reach statistical significance when compared to the conventional learning group. These results suggest that AI models like DeepSeek R1 are effective in enhancing medical undergraduates’ understanding of fundamental knowledge. However, whether they can improve students’ ability to analyze urology case summaries remains unclear. In terms of text-based questions, the DeepSeek R1 group scored 60 points, significantly higher than the control group’s 47.24 points (*P*=.02). Similarly, for image-based questions, the DeepSeek R1 group scored 6.14 points, surpassing the control group’s 4.59 points (*P*=.01). These results suggest that AI-assisted learning not only deepens medical students’ conceptual cognition but also improves their ability to interpret imaging reports, a crucial skill in clinical practice.

The survey results indicate a strong acceptance of AI-assisted learning among students. Notably, 96.19% (101/105) of respondents reported using LLMs during their undergraduate studies, highlighting the deep integration of AI tools into the daily lives of medical students. Additionally, the majority of students believe AI can effectively reduce the workload for both teachers and students, provide convenient explanations, enhance understanding of urology concepts, and stimulate learning interest. These findings suggest that students see the potential of AI in urology education and are open to using it as a supplementary learning tool. However, some students expressed concerns about the reliability and standardization of AI-assisted learning, emphasizing the need for appropriate guidance and supervision to maximize AI’s benefits while mitigating potential risks. Notably, 73.33% (77/105) of students are willing to participate in AI training provided by their institutions, highlighting the importance of integrating AI-related content into medical curricula to better prepare future health care professionals [40]. While the rise of GenAI in medical education has sparked new perspectives on traditional teaching roles [41], only 3.81% (4/105) of students believe AI will completely replace teachers, suggesting that AI is perceived as a complementary aid rather than a substitute. Additionally, 48.57% (51/105) of students believe AI could help alleviate the imbalance in educational resources, indicating that promoting AI use alongside faculty training could improve medical education, especially in underdeveloped areas. This could broaden the applicability of the study’s conclusions and benefit medical education more widely. While AI has been the subject of intensive global investigation, its practical applications in medicine remain limited [55]. The findings of this study offer empirical support to inform the future integration of AI in clinical practice and medical education. DeepSeek R1’s diagnostic accuracy of 90.48% (76/84) in urological disease-related questions, coupled with an average response time of less than 3 seconds, indicates that the model is capable of adapting to evolving medical knowledge and performing scientific reasoning. This suggests its potential for medical education, clinical decision-making [11,25], and diagnosis [56], as well as individualized treatment planning for urological tumors [26]. However, it is critical to emphasize that accuracy on standardized multiple-choice assessments does not translate to clinical validity, diagnostic reliability, or therapeutic safety in actual patient encounters. For example, resident physicians could input symptoms, signs, and imaging descriptions to instantly obtain diagnostic suggestions and recommendations for further examinations, significantly reducing misdiagnosis rates. The integration of AI into health care shows considerable promise, particularly in elevating the quality of patient management and informing diagnostic and therapeutic choices. This will facilitate the advancement of precision medicine, which has been substantiated by numerous studies to play a pivotal role in the diagnosis and management of urological diseases [57-59]. However, as AI becomes increasingly integrated, ethical, legal, and accuracy-related issues must be addressed with great care [60]. In real teaching scenarios, AI can assist educators in creating instructional materials, help students answer clinical questions, and promote interactive learning experiences [60]. For instance, instructors could use AI models to generate reference answers before case discussions and then focus on discrepancies between student responses and model outputs to provide precise feedback. Furthermore, survey results indicate that 73.33% (77/105) of students are willing to engage in AI training organized by their institutions, underscoring the potential to integrate AI literacy and critical thinking into core medical curricula [61]. Existing evidence suggests that the integration of GenAI into medical learning environments carries an acceptable safety profile, thereby supporting its further exploration. We should embrace the changes it brings to medical education with an open mindset and proactively integrate it into lifelong medical learning processes [62]. If implemented thoughtfully, GenAI has the potential to significantly support clinicians in improving medical quality, enhancing medical education, reducing workloads, and providing real-time professional knowledge support [63].

It is important to emphasize that the application of the results from this study must address the ethical considerations associated with the use of AI in medical education. From a data privacy standpoint, the training and validation of AI tools require access to large volumes of clinical and imaging data. This raises concerns related to data collection, transmission, storage, and the need to obtain informed consent [64]. If these data are not properly protected, the risk of breaches increases, underscoring the necessity of implementing stringent data protection measures and establishing clear guidelines for their usage [65]. Furthermore, attention should be paid to the authority of information and the risk of misleading. Although DeepSeek R1 demonstrated a relatively high accuracy rate in this study, AI-generated content still has the phenomenon of hallucination, meaning that the generated information may seem reasonable but is actually incorrect or misleading [66,67]. In medical education, such misinformation can mislead students, resulting in the formation of incorrect knowledge perceptions that may pose risks to future clinical decision-making. Educators must therefore assume a leading role in establishing comprehensive guidelines to govern the incorporation of AI within educational frameworks. These guidelines should foster critical thinking among students rather than passively relying on AI-generated answers [68].

This study makes distinct contributions to the existing literature. First, it simultaneously evaluates the effectiveness of 2 AI models (ChatGPT o3-mini and DeepSeek R1) in medical education, offering a more comprehensive comparison of different AI tools within the educational domain. Second, it not only examines the accuracy of AI in answering medical questions but also explores the effects of AI on students’ learning outcomes and attitudes through testing and questionnaire surveys.

However, this study also has several limitations. First, the sample size is relatively small and limited to students from a single medical college, which may limit the broader applicability of the findings. Subsequent studies are recommended to encompass larger cohorts through multicenter randomized controlled trials, thereby improving the generalizability of the findings. Second, the study duration was relatively short, and the enduring impacts of AI-assisted learning on students’ knowledge retention and clinical skill development remain unexplored. Future studies could extend the research period to better assess the enduring effects of AI in medical education. Third, this study focused solely on the field of urology within medical education. Future research could expand the scope to include other medical specialties, thereby providing more comprehensive evidence to support the broader application of AI in medical education. Fourth, the questionnaire was administered only to respondents; nonrespondents may hold less favorable views of AI. AI groups were restricted to the assigned tools without access to other search engines. Thus, observed differences may reflect variations in task structure, information format, cognitive demand, and novelty effects. For example, the conversational single-interface interaction may have been more engaging than traditional search, while the inability to cross-check AI outputs may have increased susceptibility to incorrect information. We advocate for the integration of faculty-mediated review protocols, iterative feedback loops, and mandatory AI literacy modules into urology curricula prior to broader deployment. Furthermore, we acknowledge that the DeepSeek R1 group experienced a higher attrition rate. This differential dropout raises the possibility of survivorship bias, whereby more motivated or technologically proficient students may have been more likely to complete the DeepSeek R1 intervention. Our ITT analysis under the MAR assumption supported the primary findings; however, the worst-case scenario imputation attenuated the DeepSeek R1 advantage to nonsignificance, suggesting that the result may be partially sensitive to the performance level of dropouts. Future studies should use strategies to minimize differential attrition, such as enhanced training, technical support, and reminder systems for novel AI models.

### Conclusion

ChatGPT o3-mini and DeepSeek R1 demonstrated high accuracy in answering questions related to urology. DeepSeek-assisted self-study was linked to better posttest performance than conventional online learning; by contrast, the higher scores observed with ChatGPT were only numerical and lacked statistical significance. Most students demonstrated strong receptivity to AI-assisted learning and expressed willingness to participate in AI training courses organized by their institution, suggesting that it is highly necessary to integrate AI into medical courses in the future. The findings of this study provide evidence-based references for the integration of GenAI in medical education and offer guidance for educators in formulating teaching strategies and for educational institutions in formulating policies.

## Supplementary material

10.2196/89315 Multimedia Appendix 1 R code for the statistical analyses of urology learning outcomes.

10.2196/89315 Multimedia Appendix 2 Self-reported frequency of generative AI use among participants (n=105).

10.2196/89315 Checklist 1 CONSORT-EHEALTH checklist (V 1.6.1).

## Abbreviations

CONSORT-EHEALTH: Consolidated Standards of Reporting Trials of Electronic and Mobile Health Applications and Online Telehealth.
EAU: European Association of Urology
GenAI: generative AI
ITT: intention-to-treat
LLM: large language model
MAR: missing-at-random
MCQ: multiple-choice question
NCCN: National Comprehensive Cancer Network
PASS: Power Analysis and Sample Size
